# Androgen receptor mRNA expression is a predictor for recurrence-free survival in non-muscle invasive bladder cancer

**DOI:** 10.1186/s12885-019-5512-9

**Published:** 2019-04-08

**Authors:** Masato Yasui, Takashi Kawahara, Koji Izumi, Masahiro Yao, Yukari Ishiguro, Hitoshi Ishiguro, Hiroji Uemura, Yasuhide Miyoshi

**Affiliations:** 1 Department of Urology and Renal Transplantation, Yokohama City University Medical Center, 4-57 Urafune-cho, Minami-ku, Yokohama, 232-0024 Japan; 2 Department of Urology, Yokohama City University Graduate School of Medicine, Yokohama, Japan; 3 Photocatalyst Group, Special Research Laboratory, Kanagawa Academy of Science and Technology, Kawasaki, Japan

**Keywords:** Bladder cancer, Non-muscular invasive, Androgen receptor, RT-qPCR, Recurrence

## Abstract

**Background:**

Non-muscular invasive bladder cancer (NMIBC) has a high risk of recurrence. As androgen receptor (AR) reportedly affects bladder cancer, we assessed the correlation between NMIBC recurrence and tumor *AR* expression in Japanese patients.

**Methods:**

We retrospectively reviewed 53 specimens of non-metastatic NMIBC, with recurrence-free survival (RFS) as the primary endpoint. We used real-time quantitative polymerase chain reaction to quantify *AR* mRNA expression. Kaplan–Meier product-limit estimators were used to assess RFS distribution, log-rank tests to analyze differences in RFS between high- and low-risk groups; and multivariate analyses of *AR* mRNA expression and other clinicopathological factors to predict independent factors for RFS.

**Results:**

The high *AR* mRNA-expressing group (*n* = 43) tended to have a longer median RFS (not reached) than did the low-AR group (*n* = 10; 9.04 months; *P* = 0.112). Multivariate analysis showed female sex (hazard ratio [HR]: 7.360, 95% CI: 1.649–32.856, *P* = 0.009), tumor size ≥3 cm (HR: 23.697, 95% CI: 4.383–128.117, *P* < 0.001) and low *AR* mRNA expression (HR: 0.202, 95% CI: 0.048–0.841, *P* = 0.028) to be independent predictors of shorter RFS.

**Conclusion:**

Our study showed that low *AR* mRNA expression level is an independent risk factor for RFS in Japanese patients with NMIBC. Further studies are necessary but *AR* expression might be a new indicator of recurrence of NMIBC.

## Background

Bladder cancer (BC) is a common malignancy, with an estimated 429,800 new cases and 165,100 deaths in 2012, worldwide [1]. Urothelial carcinoma is the most common type of BC, and it is stratified to non-muscular invasive bladder cancer (NMIBC) and muscular invasive bladder cancer (MIBC). Patients with MIBC have a high risk of disease progression and metastasis and usually need aggressive treatments, such as radical cystectomy or chemotherapy; whereas NMIBC has a better prognosis and can be treated curatively by transurethral resection of bladder tumor (TUR-BT). However, NMIBC has a high risk of recurrence and can progress to MIBC. To prevent recurrence and progression, intravesical instillation (IVI) of anthracycline or bacillus Calmette-Guérin (BCG) has been administered, but about 50% still recur. As the treatment has not changed for decades, new studies leading to new treatments are needed to prevent recurrence and progression.

Men tend to be diagnosed about four times more often than women [2], which implies that BC is an endocrine-related cancer. Androgen receptor (AR) signaling has been suggested to have an important role in BC occurrence and progression by previous studies [3–18].

Thus, in this study, we assessed the correlation between NMIBC recurrence and tumor AR expression in Japanese patients, by quantifying *AR* mRNA expression with real-time quantitative polymerase chain reaction (RT-qPCR).

## Methods

### Patients

We retrospectively reviewed 53 specimens of TUR-BT among 51 patients with non-metastatic NMIBC treated at Yokohama City University Medical Center and Yokohama City University Graduate School of Medicine from 2008 to 2015. In the two patients, specimens were obtained two times at the time of initial TUR-BT and at the time of recurrence. Samples for RT-qPCR came from biopsies performed during TUR-BT procedures. Both primary and recurrent NMIBC was diagnosed by TURBT. The experimental procedures were conducted in accordance with the ethical standards of the Helsinki Declaration. This study was approved by the institutional review board of Yokohama City University Medical Center. Informed consent to participate in the study were obtained from all subjects.

### Clinical assessments

The primary endpoint of this study was recurrence-free survival (RFS), which was defined as the time between the date of the initial TUR-BT and the date when the TUR for the recurrent BC was performed. Cancers detected in second-look TUR were not defined as recurrence, and RFS was counted from the first TUR-BT. Histopathological factors were assessed by pathologists specialized in uropathology.

### Real-time PCR

*AR* mRNA level was assessed by quantitative RT-qPCR method. We obtained 53 BC tissue specimens by TUR-BT to analyze gene expression. Total RNA (0.5 μg), which was isolated from the bladder tissue specimens, using Isogen (NipponGene, Tokyo, Japan), was reverse transcribed using 1 μM oligo (dT) primers (Qiagen, Germantown, MD, USA) and 4 units of Omniscript reverse transcriptase (Qiagen) in a total volume of 20 μL. Gene expression for *AR* and human glyceraldehyde-3-phosphate dehydrogenase (*GAPDH*) were examined by real-time qPCR, using *AR* primers (Applied Biosystems, Foster City, CA, USA). Gene expressions were determined using TaqMan® Gene Expression Assays (Applied Biosystems, Grand Island, NY, USA). All data were normalized to *GAPDH*; expression of one tumor was assumed to be 1 and used as reference. The 2^−ΔΔCT^ method was used to calculate relative amounts of target genes.

### Statistical analysis

The cut-off point for the *AR* mRNA level was determined by a web-based calculator cut-off finder (http://molpath.charite.de/cutoff.) and median values were used as the cut-off points for other factors to categorize continuous measurements [19]. Spearman’s rank-order correlation was used to measure the association between *AR* mRNA expression level and clinical factors. A Kaplan–Meier product-limit estimator was used to assess RFS distribution. Log-rank tests were used to analyze differences in RFS between the high- and low-risk groups. Univariate and multivariate COX models were used to analyze factors for predicting RFS. We derived relative risks and 95% confidence intervals (95% CIs). All tests were two-sided; alpha = 0.05 was considered significant. All analyses were conducted using IBM SPSS Statistics software for Windows, version 22 (IBM Corp., Armonk, NY, USA).

## Results

Patients’ characteristics are shown in Table 1. Patients’ median age at TUR-BT was 74.1 years (range: 40.8–88.8 years) and 48 (90.6%) patients were male. Of the 53 patients, 11 (20.8%) were recurrent BC and 9 patients (17.0%) were recurred within 1 year. Tumor grade was low in 39 patients (73.6%) and high in 14 patients (26.4%). Pathological T-stage was pTa in 42 patients (79.2%) and pT1 in 11 patients (20.8%). Tumors were single in 24 patients (45.3%) and multiple in 29 patients (54.7%). Tumor size was ≥3 cm in 7 patients (13.2%); concurrent carcinoma in situ (CIS) was found in 2 patients (3.8%). Immediate IVI using anthracycline was administered in 51 patients (96.2%) and additional adjuvant IVI using either BCG or anthracycline was administered in 12 patients (22.6%). Six patients (11.5%) underwent second TURs.

**Table 1:** Patients’ characteristics

Age, median (range)	74.1 (40.5–88.8)
Sex, n (%)	
Male	48 (90.6%)
Female	5 (9.4%)
Primary or recurrence, n (%)	
Primary	42 (79.2%)
Recurrence	11 (20.8%)
Recurrence within one year, n (%)	
Yes	9 (17.0%)
No	44 (83.0%)
Tumor grade, n (%)	
Low	39 (73.6%)
High	14 (26.4%)
T stage, n (%)	
pTa	42 (79.2%)
pT1	11 (20.8%)
Number of tumor, n (%)	
Single	24 (45.3%)
Multiple	29 (54.7%)
Tumor size, n (%)	
< 3 cm	46 (86.8%)
≥ 3 cm	7 (13.2%)
Concurrent CIS, n (%)	
Yes	2 (3.8%)
No	51 (96.2%)
Immediate intravesical chemotherapy, n(%)	
Yes	51 (96.2%)
No	2 (3.8%)
Additional adjuvant intravesical chemotherapy, n (%)	
Yes	12 (22.6%)
No	41 (77.4%)
Second TUR, n (%)	
Yes	6 (11.5%)
No	47 (88.5%)

*CIS* carcinoma in situ, *TUR* transurethral resection

We found no significant association between *AR* mRNA expression level and other clinicopathological factors (Spearman’s rank-order correlation; Table 2).

**Table 2:** Spearman’s rank-order correlation between each variables

	Sex	Age	T stage	Tumor grade	Tumor size	Number of tumor	Recurrence/Primary	Instillation	AR mRNA expression
Sex	1.000	0.071	−0.006	0.099	−0.126	0.034	0.153	−0.020	−0.174
Age	0.071	1.000	0.056	−0.160	0.063	−0.093	0.056	0.010	−0.009
T stage	−0.006	0.056	1.000	0.432	0.075	0.372	−0.262	0.057	−0.110
Tumor grade	0.099	−0.160	0.432	1.000	0.019	0.373	−0.201	0.392	−0.149
Tumor size	−0.126	0.063	0.075	0.019	1.000	−0.093	−0.200	−0.078	0.188
Number of tumor	0.034	−0.093	0.372	0.373	−0.093	1.000	−0.095	0.492	−0.051
Recurrence/primary	0.153	0.056	−0.262	−0.201	0.200	−0.095	1.000	0.057	−0.110
Instillation	−0.020	0.010	0.057	0.392	−0.078	0.492	0.057	1.000	−0.085
AR mRNA expression	−0.174	−0.009	−0.110	−0.149	0.188	−0.051	−0.110	−0.085	1.000

*mRNA* messenger ribonucleic acid

**Fig. 1:**
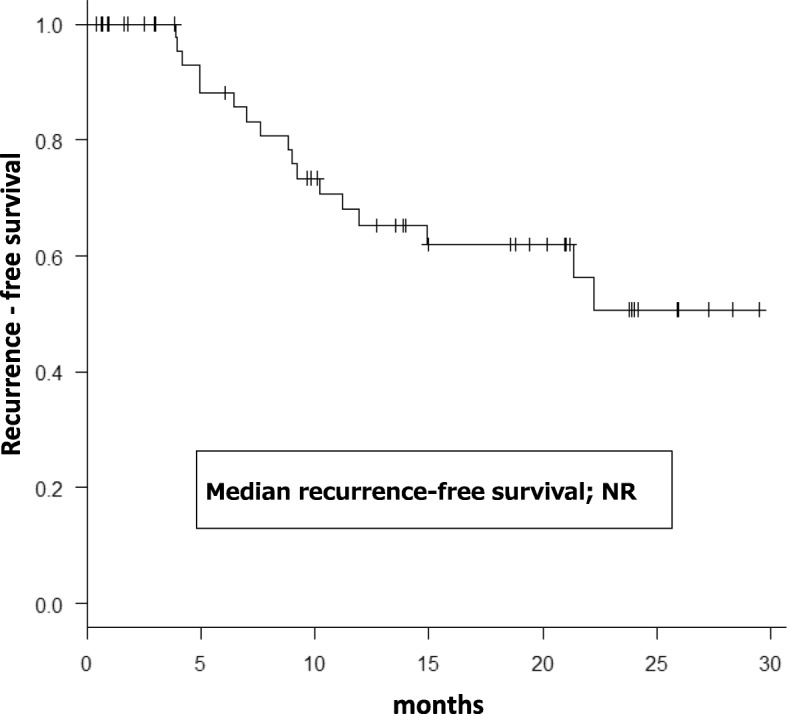
Kaplan–Meier curve for RFS after TUR-BT. Within a median observation period of 10.2 months (range: 0.4–29.5 months), recurrence occurred in 17 patients (32.1%)

The Kaplan–Meier curve for RFS is shown in Fig. 1. Within a median observation period of 10.2 (range: 0.4–29.5) months, recurrence occurred in 17 patients (32.1%).

**Fig. 2:**
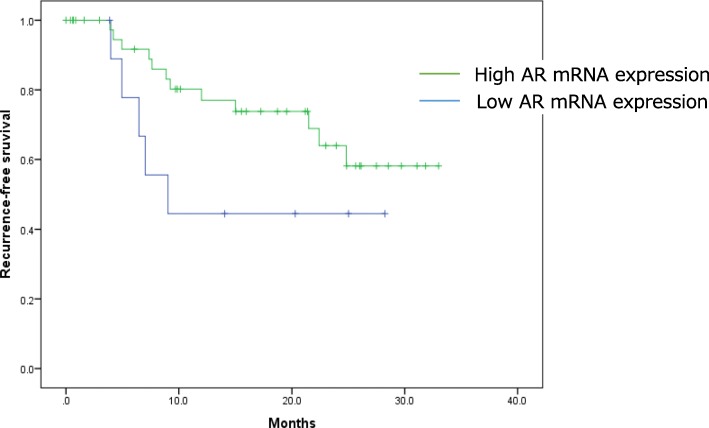
Kaplan–Meier curve for recurrence-free survival (RFS), stratified by *AR* mRNA expression level. Green line: RFS curve for high *AR* mRNA expression level group; blue line: RFS for low *AR* mRNA expression level group. The high *AR* mRNA-expressing group tended to have a longer median RFS (not reached) than did the low-*AR* group (9.04 months; *P* = 0.112)

We stratified the 53 patients into two groups: 43 in the high *AR* mRNA-expressing group and 10 in the low *AR* group. The high *AR* group showed a tendency for longer median RFS (not reached) than did the low-*AR* group (9.04 months, *P* = 0.112; Fig. 2).

Results of univariate and multivariate analyses to identify risk factors for RFS are shown in Table 3. In univariate analysis, female sex (hazard ratio [HR]: 5.009, 95% CI: 1.589–15.787, *P* = 0.006) and tumor size ≥3 cm (HR: 3.487, 95% CI: 1.282–9.480, *P* = 0.0141) were significantly related to shorter RFS. Multivariate analysis showed that female sex (HR: 7.360, 95% CI: 1.649–32.856, *P* = 0.009), tumor size ≥3 cm (HR: 23.697, 95% CI: 4.383–128.117, *P* < 0.001) and low *AR* mRNA expression (HR: 0.202, 95% CI: 0.048–0.841, *P* = 0.028) were independent predictors of shorter RFS.

**Table 3:** Univariate and multivariate analyses for predicting recurrence-free survival

		Univariate				Multivariate			
		*p* value	HR	HR 95% CI		*p* value	HR	HR95% CI	
Sex	Women vs men	0.006	5.009	1.589	15.787	0.009	7.360	1.649	32.856
Age	High vs low	0.324	1.633	0.617	4.325	0.453	0.640	0.199	2.054
T stage	pT1 vs pTa	0.925	1.056	0.342	3.261	0.096	5.274	0.743	37.435
Tumor grade	High vs low	0.526	0.694	0.225	2.142	0.657	0.650	0.097	4.357
Tumor size	≥3 cm vs < 3 cm	0.014	3.487	1.282	9.480	< 0.001	23.697	4.383	128.117
Number of tumor	Multiple vs single	0.189	0.527	0.203	1.370	0.477	1.688	0.398	7.148
Tumor recurrence	Recurrence vs primary	0.531	1.397	0.491	3.973	0.052	4.122	0.988	17.190
Instillation	Yes vs no	0.068	0.253	0.058	1.108	0.088	0.182	0.026	1.290
AR mRNA expression	High vs low	0.123	0.438	0.153	1.250	0.028	0.202	0.048	0.841

*mRNA* messenger ribonucleic acid, *HR* hazard ratio, *CI* confidence interval

## Discussion

Although the function of AR signaling in BC is unclear, it is known to play important roles in prostate cancer occurrence and progression, and reportedly affects kidney, lung, breast and liver cancers [20].

AR is a nuclear steroid hormone receptor, composed of several domains, N-terminal transactivation domain, DNA binding domain, a hinge region and a ligand-binding domain (LBD). In the cytoplasm, heat shock protein-90 (HSP90) binds with the LBD. As 5α-dihydrotestosterone binds to AR, AR releases HSP90 and translocates into the nucleus, binds to the androgen-response element, and promotes gene transcription [3–6, 21].

AR signaling and the urinary tract may be critically associated. Studies in animal models have shown AR to be present in several tissues in lower urinary tract and may affect growth, differentiation and maintenance of urinary bladder tissue [6, 7, 22, 23].

Reportedly, AR signaling has a pivotal role in BC occurrence and progression. Testosterone increased the risk of N-butyl-N-(4-hydroxybutyl) nitrosamine (BBN)-induced bladder tumors in female rats, whereas diethylstilbestrol, a synthetic form of female hormone estrogen, inhibited incidence in male rats [8]. Also, intact female rats administered with testosterone had higher incidences of both bladder calculi and tumors, but oophorectomized rats did not, which implies that testosterone in combination with estrogen may contribute to BC formation [9]. Moreover, flutamide, a nonsteroidal antiandrogen therapy, inhibited BBN-induced bladder tumor incidence in rats, whereas finasteride, a 5α-reductase inhibitor, did not; this suggests that testosterone itself has an effect in bladder carcinogenesis but its converting form, 5α-dihydrotestosterone, does not [10].

Studies with animal models have indicated that AR has an important role in BC. Miyamoto et al. found that both male and female *AR*-knockout mice (ARKO) did not develop BBN-induced tumors, which suggests that AR has a crucial activity in bladder carcinogenesis [11]. That study also found that 25% of ARKO mice administered with dihydrotestosterone (DHT) developed tumors, compared with 50% of castrated male wild-type rats and 92% of intact wild-type male rats. These findings suggest that absence or low levels of androgen could restore AR signaling, or that androgens could induce bladder carcinogenesis independently of AR [7, 24]. Another study showed that mice lacking AR only in the urothelial tissues had lower incidence of BBN-induced BC and a higher survival rate than wild-type mice [12].

Therefore, the association between BC and AR has been the focus of several recent studies. Expression of AR have indicated to have high risk in bladder cancer occasion, but as for recurrence, expression of AR have shown to contribute to longer RFS. An immunohistochemical study of 169 patients indicated that recurrence was less likely for patients with AR^+^ BC specimens [13]; another study showed that loss of AR was strongly associated with higher grade and more invasive tumors [14]. A study of the predictive value of *AR* mRNA expression in pT1 NMIBC showed that high *AR* mRNA expression was an independent predictor for longer RFS and cancer-specific survival [15], which was similar to our present findings that high *AR* mRNA level was an independent predictor for RFS in NMIBC (including pTa and pT1).

Reportedly, AR pathway inhibitors have been shown to be effective in preventing BC recurrence. In patients with double primary cancers of the prostate and the bladder, androgen-deprivation therapy (ADT) to treat their prostate cancers reduced the risk of recurrence of AR^+^ BC [16]. Enzalutamide, an AR-signaling inhibitor, is also reported to inhibit AR^+^ BC cell growth in vivo [17]. BCG IVI has been the most effective therapy for NMIBC; its mechanism may be due to interaction between BCG and the AR pathway. DHT down-regulates NF-κB-mediated IL-6 expression, whereas AR blockers inhibit the effect of DHT. This result suggests that AR pathway inhibitors could improve the efficacy of BCG treatment [18].

As described above, previous clinical studies demonstrated that higher AR expression in both mRNA levels and protein levels were correlated with favorable outcome in NMIBC [13–15]. Moreover, our current study also demonstrated that higher AR mRNA expression levels were associated with longer recurrence-free survival in NMIBC. On the other hand, enhanced AR signaling pathway could play an important role in cancer initiation and progression in vitro and in vivo. There were discrepancies between our findings and previous reports from studies in vitro or in vivo [8, 10–12]. Even though the reasons for discrepancies are unknown, several possible hypothesis have been suggested. It has to be considered that AR expression, and thus the role of AR, might change during the progression of bladder cancer, as indicated by the previously cited studies [14]. In addition, Sikic et al. reported that different AR subtype (AR 1 and AR2) has different role in bladder cancer carcionogenesis [14].

The mechanism of AR signaling in BC is still unclear, therefore, further studies of the AR pathway in NMIBC are needed.

Our study had several limitations. First, it was a retrospective study with relatively few patients and a short follow-up period. Second, CIS was not analyzed in this study. CIS is an essential risk factor of recurrence and progression in NMIBC, but our study cohort had only two BCs with coexisting CIS. Finally, for verify the role of AR pathway in NMIBC, not only AR mRNA expression levels, but also protein expression levels should be investigated in current study. Future clinical studies would need to estimate an appropriate sample size to include enough of these patients and investigate mRNA and protein expression levels simultaneously.

## Conclusions

Our study show that *AR* mRNA expression is an independent predictor for RFS in Japanese patients with NMIBC. The AR pathway is now attracting attention in BC research, and may provide new methods to treat bladder cancer and prevent its recurrence.

## Abbreviations

AR: Androgen receptor
ARKO: AR-knockout (mice)
BBN: N-butyl-N-(4-hydroxybutyl) nitrosamine
BC: Bladder cancer
BCG: Bacillus Calmette-Guérin
CIS: Carcinoma in situ
DHT: Dihydrotestosterone;
HR: Hazard ratio
HSP90: Heat shock protein-90
IVI: Intravesical instillation
LBD: Ligand-binding domain
MIBC: Muscular invasive bladder cancer
NMIBC: Non-muscular invasive bladder cancer
RFS: Recurrence-free survival
RT-qPCR: Real-time quantitative polymerase chain reaction
TUR-BT: Transurethral resection of bladder tumor
XIST: X-inactive specific transcript
